# Window of Opportunity: Rate of Referral to Infertility Providers among Reproductive-Age Women with Newly Diagnosed Gynecologic Cancers

**DOI:** 10.3390/jcm13164709

**Published:** 2024-08-11

**Authors:** Emily H. Frisch, Meng Yao, Hanna Kim, Olivia Neumann, Danielle B. Chau, Elliott G. Richards, Lindsey Beffa

**Affiliations:** 1 ObGyn and Women’s Health Institute, Cleveland Clinic, Cleveland, OH 44124, USA; 2 Quantitative Health Sciences, Cleveland Clinic, Cleveland, OH 44195, USA; 3 Department of Reproductive Endocrinology and Infertility, Cleveland Clinic, Cleveland, OH 44106, USA; 4 Department of Gynecology Oncology, Cleveland Clinic, Cleveland, OH 44195, USA; 5 Virginia Oncology Associates, Norfolk, VA 23502, USA

**Keywords:** oncofertility, gynecologic cancer, REI referrals

## Abstract

**Background/Objective:** Fertility preservation is an important part of oncologic care for newly diagnosed gynecologic cancers for reproductive-age women, as many treatment options negatively impact fertility. The goal of this study is to examine factors that influence access to fertility specialists for women with newly diagnosed gynecologic cancer. **Methods:** This institutional review board approved a retrospective cohort study investigating the impacting factors on the referral rate from gynecologic oncologists (GO) to reproductive endocrinologists and infertility (REI) specialists at a single academic institution between 2010–2022 for patients age 18–41 at diagnosis. Electronic medical records were used to identify demographics and referral patterns. Mixed logistic models were utilized to control cluster effects of the physicians. **Results:** Of 816 patients reviewed, 410 met the criteria for inclusion. The referral rate for newly diagnosed gynecologic malignancies was 14.6%. Younger patients were more likely to have an REI referral (*p* < 0.001). The median time from first GO visit to treatment was 18.5 days, and there was no significant difference in those who had REI referrals (*p* = 0.44). Only 45.6% of patients had fertility desire documented. A total of 42.7% had fertility-sparing treatment offered by a GO. REI referral did not significantly change the time to treatment (*p* = 0.44). An REI referral was more likely to be placed if that patient had no living children, no past medical history, or if the referring GO was female (OR = 11.46, 6.69, and 3.8, respectively). **Conclusions:** Fertility preservation counseling is a critical part of comprehensive cancer care; yet, the referral to fertility services remains underutilized in patients with newly diagnosed gynecologic cancer. By demonstrating these biases in REI referral patterns, we can optimize provider education to enhance fertility care coordination.

## 1. Introduction

The field of oncofertility has grown exponentially in the last two decades, with the term first being coined in 2006 [1]. The American Society of Reproductive Medicine recommends that providers discuss the risk of infertility in all patients treated for cancer during their reproductive years and that patients be informed of options before cancer treatment [2]. Yet, studies have shown that fertility preservation education following a cancer diagnosis is an unmet need for reproductive-age women [3]. Reassuringly, for appropriately selected patients, survival outcomes do not appear to differ significantly between radical and fertility-sparing approaches in gynecologic cancer therapies [4,5]. These studies encompass more than one cancer system, often grouping breast, hematologic, lung, and occasionally gynecologic organ systems together to evaluate fertility preservation pursuits. While guidelines recommend referral for possible fertility preservation or counseling, the rate of referrals remains low in the literature (1–20.6%) [6,7].

There are many patient-reported barriers to fertility preservation in the setting of oncologic diagnoses, including lack of education, stress of cancer diagnosis, and financial burden [8]. These barriers can often be traced back to a lack of referral to a fertility specialist or provider who is knowledgeable about fertility-sparing options. Financial barriers can, in many cases, be overcome with insurance appeals and grants, yet without a fertility preservation consult, patients may not have the opportunity to learn about these options. Tellingly, studies that address cancers in the reproductive-age population have shown that patients are more likely to elect fertility preservation if they receive a consult [9].

Fertility preservation is an important, yet often overlooked, consideration in the setting of new early-stage gynecologic cancers for reproductive-age women. About 10% of the 1.3 million women living with gynecologic cancer are younger than 50 years old [4]. A discussion about fertility preservation and fertility-sparing therapies is imperative, especially as childbearing is being increasingly postponed [10]. Many studies have commented on the safety and efficacy of fertility-sparing options for women of reproductive age with a gynecologic cancer [11]. However, only 5.5% of reproductive-age women with lung, breast, colorectal, or cervical cancer in the U.S. have been evaluated for fertility preservation [11]. Only 25.5% of cancer survivors reported meeting their desired family size before a cancer diagnosis [12]. Oncologic care can lead to irreversible ovarian damage from gonadotoxic chemotherapy, radiation, and/or surgery [13,14]. In the advent of check point inhibitor immunologic therapies for cancer treatment, there is not a complete understanding of the extent of their impact on fertility. However, they have been demonstrated to impair follicular reserve and ability of oocytes to mature and ovulate [15]. Women of reproductive age who are diagnosed with gynecologic cancer deserve timely counseling on the risks and benefits of fertility-sparing versus conventional medical and surgical therapies. REI providers can offer patients with cancer in this setting surgical preservation (i.e., ovarian transposition), medication preservation, oocyte preservation, embryo preservation, or ovarian tissue preservation. The timing and preference is patient-specific [16,17]. In these fertility consultations, the patient has the opportunity to learn and discuss their options.

Fertility preservation options, pregnancy outcomes, and referral rates in the setting of breast cancer in reproductive-age women have been very well described [16,18,19]. However, there is a paucity of data on the referral and attendance rates to fertility specialists for women of reproductive age with new gynecologic cancer diagnoses. Fertility preservation is an important part of oncologic care for newly diagnosed gynecologic cancers for reproductive-age women. Implicit biases held by gynecologic oncologic (GO) physicians can unintentionally impact practice patterns related to referrals for fertility preservation [20].

The goal of this study was to examine factors that influence access to fertility specialists for women with newly diagnosed gynecologic cancer. Our primary aim was to examine the rate of referral to fertility services for women with newly diagnosed gynecologic cancer and factors that impact referral rates. Our secondary aims were to examine the rate of women with infertility referrals presenting for initial infertility consultations and undergoing workup, as well as the time from initial referral to consultation, the rate of women with newly diagnosed gynecologic cancer undergoing fertility preservation care, and the fertility outcomes for those who underwent fertility preservation.

## 2. Materials and Methods

This institutional review board-approved, retrospective cohort study investigated the impacting factors on the referral rate from GOs to reproductive endocrinologists and infertility (REI) specialists at a single academic institution between 2010 and 2022. The patients included were aged 18–41 at the time of gynecologic cancer diagnosis to encompass women eligible for fertility preservation according to institution-based age criteria. Patients were excluded if they had had a prior hysterectomy, received GO care at outside institutions, or had prior cancer diagnoses. These records were sorted through an initial chart query for the inclusion criteria listed above, which yielded 816 patients.

Data were abstracted from electronic medical records (EMR, Epic Systems©, Verona, WI, USA). Descriptive statistics were performed to describe referral rates, treatment plans, and fertility visit outcomes. Our institution’s EMR has only two procedure codes that can be used for referring a patient to fertility services. Therefore, a referral from GO to REI was determined to have occurred when there was one of the following orders placed in the patient’s chart: “Consult to Infertility Clinic” (procedure code 2115327) or “Consult to Fertility/Cancer Patient” (procedure code 2131187).

Variables that were included were age at diagnosis, BMI, living children at time of diagnosis, and year of diagnosis. Demographic variables that were included were as follows: race, ethnicity, and marital status. Past surgical history; past medical history; and, more specifically, pelvic procedure history were reviewed. For GO information, the following variables were included: the type of cancer, GO’s gender, GO’s years in practice, and time between contact and treatment. The GO office visit note was queried for documentation of the patient’s desire for future fertility and the selected fertility-sparing treatment options. The REI office visit note was reviewed for documentation of fertility treatments, fertility testing, fertility preservation options, and pregnancy.

Continuous measures that showed departure from normality were summarized using medians and quartiles and compared using Wilcoxon rank sum tests. Categorical factors were summarized using frequencies and percentages and were compared using Pearson’s chi-square tests or Fisher’s exact tests. Mixed logistic models were utilized to predict REI referral, controlling clinical factors and cluster effects of physicians. All analyses were conducted using SAS (version 9.4, The SAS Institute, Cary, NC, USA), and a *p* less than 0.05 was considered statistically significant.

## 3. Results

### 3.1. Demographics

Of the 816 patients reviewed, 410 met the criteria for inclusion. Of the patients with newly diagnosed gynecologic malignancies, 14.6% were referred to REI. In addition, 50.2% of patients were nulliparous and 43.9% were aged 18–34 (Table 1). There was no significant difference in the race (*p* = 0.22) or ethnicity (*p* = 0.65) of those who received referrals to REI providers. Younger patients were significantly more likely to have an REI referral (*p* < 0.001). Women who were married were more likely to have a referral (*p* = 0.019). Those who had type 2 diabetes were less likely to receive an REI referral (*p* = 0.031). Those who had a BMI greater than 30 were significantly less likely to receive an REI referral (*p* = 0.012). Past medical history of hypertension, asthma, kidney disease, or GI disease did not have significant associations with the referral. If a patient had no past medical history, they were significantly more likely to receive an REI referral (*p* < 0.001). Prior pelvic surgery was not significantly associated with receiving an REI referral (*p* = 0.084) (Table 1).

### 3.2. Cancer Types

Of the patients included in the study, 37.1% had cervical cancer, 24.6% had ovarian cancer (epithelial, peritoneal, tubal), 20.2% had uterine cancer, 6.3% had gestational trophoblastic neoplasm, 6.1% had borderline ovarian tumors, 3.7% had vaginal/vulvar cancer, 2.7% had sex cord stromal/germ cell tumors, and 1.2% had other types of ovarian cancer like neuroendocrine tumors (Table 2). There were 418 total cancer types in 410 patients, as some patients had 1 more than one type of gynecologic cancer. Those who had ovarian cancer (epithelial, peritoneal, or tubal) were significantly more likely to have an REI referral compared to other gynecologic cancers (*p* = 0.044).

### 3.3. Gynecologic Oncology Providers

Of the 12 GO providers at the single institution during the years 2010–2022, 25% were female providers. Female gynecologic oncologists were significantly more likely to refer newly diagnosed gynecologic cancer patients to REI (*p* = 0.010). The included GO providers had been in practice for 7 to 33 years. Those who had started their GO practice more recently were more likely to place an REI referral (*p* = 0.032) (Table 2).

### 3.4. Treatment Types

Of the total of 410 people with newly diagnosed gynecologic malignancy, the first steps in oncologic therapy were surgery (84.4%), chemotherapy (5.6%), chemotherapy with radiation (6.7%), and radiation alone (1.7%). In those who had an REI referral, the first steps in oncologic therapy were surgery (91.7%), chemotherapy (5.6%), chemotherapy with radiation (8.3%), and radiation alone (0%). The median time from diagnosis to treatment was 18.5 days IQR: [8.0,35.0], which was not significantly different in those who had an REI referral compared to those who did not (*p* = 0.44).

Those who had an REI referral were significantly more likely to receive fertility-sparing surgery (*p* < 0.001). Of those who had an REI referral, 71.7% underwent fertility-sparing treatment (Table 2).

### 3.5. Documentation

Only 45.6% of patients had a desire for future fertility documented in their GO consult note, and 54.4% of new GO consultations did not have documentation of a desire for future fertility. Of those who had consult notes with documented fertility specifications, 32.7% desired future fertility at the time of GO consultation.

### 3.6. Attendance and Pregnancy

The attendance rate was 81.7% for REI referrals. Fertility options were offered at 91.8% of REI visits (Table 3). A total of 62.2% of patients elected to undergo fertility testing, and 46.7% underwent fertility preservation (Table 3). With a median interval of 46 months (3.8 years), 4.4% of patients who received fertility-sparing surgery and received REI referrals achieved pregnancy (Table 4).

### 3.7. Mixed Model

In the mixed-effect model, REI referrals were more likely to be placed in patients who had no living children (OR: 11.46, *p* = 0.004), no past medical history (OR: 6.69, *p* = 0.007), or for whom the referring GO physician was female (OR: 3.80, *p* = 0.043) (Table 5).

## 4. Discussion

This study highlights that low referral rates are a barrier for patients who might otherwise receive fertility care. We found a high attendance rate for the fertility consults once a referral was placed and that a significant majority of patients elected to undergo further workup and treatment following consultation. This referral rate from GO to REI is markedly lower than previously studied referral rates from all oncology providers to REI, which are cited as 1–20.6% in the literature [6,7].

The REI referral rate and documented fertility desire in our cohort were lower than expected despite presumably well-intentioned oncologists. The notable differences included that those who did not have any living children were significantly more likely to have an REI referral placed. This means that younger, nulliparous, and single women were more likely to receive REI referrals after a gynecologic cancer diagnosis. We investigated whether medical history was associated with referral patterns, but a significant difference was only seen in those who had type 2 diabetes, obesity, and mental health illnesses. BMI has been shown in the literature to negatively impact referral; of note, many REI practices have BMI cutoffs for assisted reproductive technology treatments ranging from 35 to 50 [21,22]. These serve as potential barriers that negatively impact the referral rate. Race and ethnicity did not negatively impact the referral rate to REI. 

The majority of GO providers during the 12-year retrospective review were male. The female GO providers were significantly more likely to place referrals compared to male GO providers. The female GOs were also more recently out of training, which could have contributed towards improved REI referral rates. Previously, it has been shown that female oncologists refer patients (with all cancer types) to REI specialists at a greater rate than male oncologists [23]. Here, it is shown that the odds ratio for female oncologists to place an REI referral is 2.9. This current study’s findings also demonstrate that female GO providers place REI referrals significantly more, at an odds ratio of 3.80, controlling for other factors. The start date and the GO’s years in practice were studied to assess the trend in referrals over the 12 years. Those who had more recently completed GO training were more likely to place an REI referral. 

A variety of gynecologic cancers were seen in the reproductive age population, with the most common being cervical cancer, followed by ovarian cancer, then borderline ovarian tumors. This is consistent with the literature, which demonstrates the most common cancer in reproductive-age women is cervical cancer [24,25]. The first definitive treatment in the treatment plan was most commonly surgery in those with/without REI referrals compared to chemotherapy, radiation, or chemotherapy plus radiation. When performed with fertility in mind, surgery has the greatest potential to preserve fertility compared to therapies like gonadotoxic chemotherapy and radiation to the ovary/uterus.

Fertility-sparing treatment was offered to 42.7% of all patients in this study regardless of REI referral. Notably, those who were offered fertility-sparing surgery were also more likely to receive an REI referral. Many gynecologic cancers can be treated with fertility-sparing options and may not necessitate a referral to REI. However, gynecologic surgeries (even conservative, ovary-sparing surgeries) have potential to impact future fertility, and it is not unreasonable to consider fertility consultation when starting any form of oncologic therapy so that patients’ fertility goals can better be addressed. The American Society of Clinical Oncology recommends this as part of education and informed consent before cancer therapy, “oncologists should address the possibility of infertility to patients treated during their reproductive years and be prepared to discuss possible fertility preservation options to refer appropriate and interested patients to reproductive specialists” [26]. An oncology consultation is a highly involved discussion. It does not necessitate comprehensive counseling on fertility, but it begins a discussion that REI providers can continue with the patient. GO providers are well-intentioned, and their time during patient visits is focused on cancer workup and treatment; however, it is an important opportunity to initiate a discussion about fertility, which is part of holistic cancer care for reproductive-age women. 

The majority of patients in this study were offered surgical intervention as an initial treatment of their cancer. The majority of these surgical interventions can reduce or eliminate future fertility. These findings highlight the need for timely referral to REI prior to surgery. With a median time to treatment of 18.5 days in all patients with or without REI referrals, fertility preservation referral appears to have a minimal impact on standard oncologic care. Given that the difference in time from gynecologic cancer diagnosis to oncologic intervention is not clinically significant whether fertility referral occurs or not, this is a brief window of opportunity for offering fertility preservation without impacting oncologic care or overall survival. By identifying these shortfalls in REI referral patterns, we can address barriers and optimize provider education to enhance fertility care coordination.

Importantly, there was no documentation of fertility goals by GO providers for 54.3% of new GO visits. The initial encounter with a GO provider is an important opportunity to begin a dialogue regarding a new cancer diagnosis and future fertility, and it is critical to document that these conversations have taken place. Our findings demonstrate a need to enhance discussion and documentation regarding fertility, especially when it pertains to potential therapies that directly impact fertility. 

The attendance rate for REI referrals, when placed, were high (81.7%). Most patients who attended this consultation were offered fertility treatment, and of those, the majority underwent fertility testing and fertility preservation. Of the 14.5% of women who had an REI referral after a gynecologic malignancy diagnosis, 46.7% of those women underwent fertility preservation, indicating a high utilization of fertility preservation options. Utilization rates have varied in the literature. In a study of 70 European infertility centers, only 7.6% of breast cancer patients referred to fertility specialists before chemotherapy initiation underwent fertility preservation [27]. However, in a US study that looked at breast cancer, 58.4% underwent fertility preservation, which reflects the rate of utilization in this study [28]. In a more recent study, fertility preservation utilization was 3.1% to 8.7% for oocytes and 9% to 22.4% for embryos [29]. Our current study’s utilization of fertility preservation is higher than that of previously reported values. However, with referral to REI occurring only 14.6% of the time in this study, this demonstrates a large potential loss of women that may have benefitted from fertility preservation options if a referral had been placed.

To our knowledge, this is the first study to evaluate GO-to-REI referral rates. Overall, this study demonstrates that the window of opportunity for referral to REI is often underutilized and that there is a high utilization of fertility services when an REI referral is placed. 

Practice patterns vary widely by region and health system, and so these results have limited generalizability. For example, the patient population for this study was in a state that does not mandate financial coverage for fertility. This study does not investigate the impact of financial burden that can be associated with fertility preservation. Fertility costs vary greatly between insurances. Cost and access are important considerations that should be addressed in future studies. 

We plan to prospectively investigate the rate of referral from gynecologic oncologists to fertility specialists at a major academic institution where the gynecologic oncology and fertility departments have robust, large volume practices. This will allow for a better understanding of barriers to fertility care and demonstrate how to optimize outcomes, patient education, and access to fertility care in women with new early-stage gynecologic cancers.

### Limitations

We recognize that this study focuses on GO-to-REI referrals and does not encompass other cancer types. Prior work has looked at REI referral rates in oncology generally [6]. In contrast, this study highlights gynecologic cancers given the fact that GO providers are also gynecologists and these cancer types directly impact the reproductive organs. Future studies are needed to further develop our understanding as providers to enhance the referral process for REI in a timely manner. 

Immunotherapy was not utilized in this patient population during the included years, and therefore is not included in Table 2. We recognize the importance of this treatment adjuvant and hope to include it in future studies.

This studies highlights GO visit chart documentation of fertility preservation discussions. Off-record discussions are likely taking place between patients and providers, and the fact that we cannot capture or measure such discussions arguably poses a limitation to the study. While off-record discussions limit our ability to gauge the amount of fertility preservation communication taking place, our study results still highlight that fertility preservation is, even in that best-case scenario of robust off-record discussions, not enough of a priority in GO providers to warrant a thorough documentation. 

Given the limitations of chart reviews and inconsistencies in our EMR, which affect our ability to accurately and consistently describe these attributes and variables, we chose not to include variables such as socio-economic factors and mental health status. In future prospective studies and quality improvement projects, we hope to address this by developing automatic alerts in our EMR to recommend REI referral when a reproductive-age woman has a new cancer diagnosis. 

## 5. Conclusions

Fertility preservation counseling is a critical part of comprehensive cancer care; yet, the rate of referral to fertility services remains understudied in patients with newly diagnosed gynecologic cancer. This study emphasizes the importance of GO training and exposure to fertility issues as a result of cancer therapy. Fertility preservation is a critical component, and this study highlights the opportunities for quality improvement through protocols and guidelines.

Here, we show that, while there is a window of opportunity for fertility services after diagnosis of gynecologic cancer, referral rates are only 14.6% in those with newly diagnosed gynecologic malignancies.

## Figures and Tables

**Table 1:** Demographics of newly diagnosed GO without REI referral vs. REI.

Factor	Total (N = 410)	No Referral (N = 350)	Referral Placed (N = 60)	*p*-Value
Age at diagnosis (median) IQR (when was first abnormal imaging or biopsy found)	35.0 [31.0, 39.0]	36.0 [32.0, 39.0]	32.0 [28.5, 35.5]	<***0.001*** ^b^
Age at Diagnosis n (%)				<***0.001*** ^c^
18–34	180 (43.9)	139 (39.7)	41 (68.3)	
35–41	230 (56.1)	211 (60.3)	19 (31.7)	
Living children > 0				<***0.001*** ^c^
No	204 (49.8)	156 (44.6)	48 (80.0)	
Yes	206 (50.2)	194 (55.4)	12 (20.0)	
Race				0.22 ^d^
White	327 (79.8)	277 (79.1)	50 (83.3)	
African American	61 (14.9)	56 (16.0)	5 (8.3)	
Asian	8 (2.0)	6 (1.7)	2 (3.3)	
Multiracial/Multicultural	14 (3.4)	11 (3.1)	3 (5.0)	
Ethnicity				0.65 ^d^
Not Hispanic	385 (93.9)	329 (94.0)	56 (93.3)	
Hispanic	13 (3.2)	10 (2.9)	3 (5.0)	
Unknown	12 (2.9)	11 (3.1)	1 (1.7)	
Marital Status at time of diagnosis				***0.019*** ^d^
Married	198 (48.3)	178 (50.9)	20 (33.3)	
Single	198 (48.3)	158 (45.1)	40 (66.7)	
Widowed	5 (1.2)	5 (1.4)	0 (0.00)	
Unknown	9 (2.2)	9 (2.6)	0 (0.00)	
Med hx: type 2 diabetes	37 (9.0)	36 (10.3)	1 (1.7)	***0.031*** ^c^
Med hx: hypertension	60 (14.6)	54 (15.4)	6 (10.0)	0.27 ^c^
Med hx: asthma	33 (8.0)	27 (7.7)	6 (10.0)	0.61 ^d^
Med hx: kidney disease	4 (0.98)	4 (1.1)	0 (0.00)	0.99 ^d^
Med hx: GI disease	20 (4.9)	20 (5.7)	0 (0.00)	0.056 ^d^
Med hx: obesity (BMI > 30)	148 (36.1)	135 (38.6)	13 (21.7)	***0.012*** ^c^
Med hx: mental health illness	76 (18.5)	72 (20.6)	4 (6.7)	***0.010*** ^c^
Med hx: other malignancy	13 (3.2)	11 (3.1)	2 (3.3)	0.99 ^d^
Med hx: none	170 (41.5)	132 (37.7)	38 (63.3)	<***0.001*** ^c^
Pelvic hx: ovarian cystectomy	19 (4.6)	12 (3.4)	7 (11.7)	***0.012*** ^d^
Pelvic hx: none	255 (62.2)	217 (62.0)	38 (63.3)	0.84 ^c^

Statistics are presented as median [P25, P75], N (column %). *p*-values: b = Wilcoxon Rank Sum test, c = Pearson’s chi-square test, d = Fisher’s exact test.

**Table 2:** Oncologic factors of newly diagnosed GO without REI referral vs. with REI referral.

Factor	Total (N = 410)	No Referral (N = 350)	Referral Placed (N = 60)	*p*-Value
Cancer: Cervical	152 (37.1)	134 (38.3)	18 (30.0)	0.22 ^c^
Cancer: Epithelial/Ovarian/Peritoneal/Tubal	101 (24.6)	80 (22.9)	21 (35.0)	***0.044*** ^c^
Borderline ovarian tumor	25 (6.1)	14 (4.0)	11 (18.3)	<***0.001*** ^c^
Cancer: Sex cord stromal/germ cell	11 (2.7)	10 (2.9)	1 (1.7)	0.60 ^c^
Cancer: Gestational trophoblastic	26 (6.3)	24 (6.9)	2 (3.3)	0.30 ^c^
Cancer: Uterine	83 (20.2)	75 (21.4)	8 (13.3)	0.15 ^c^
Cancer: Vaginal/vulvar	15 (3.7)	15 (4.3)	0 (0.00)	0.10 ^c^
Cancer: Other ovary (i.e., neuroendocrine)	5 (1.2)	5 (1.4)	0 (0.00)	0.35 ^c^
Gyn Onc gender				***0.010*** ^c^
female	80 (19.5)	61 (17.4)	19 (31.7)	
male	330 (80.5)	289 (82.6)	41 (68.3)	
Gyn Onc Start Year	2006 [1989, 2015]	2004 [1989, 2015]	2010 [2004, 2015]	***0.032*** ^b^
Desire for future fertility at time of gyn onc visit?				<***0.001*** ^c^
yes	134 (32.7)	77 (22.0)	57 (95.0)	
no	53 (12.9)	53 (15.1)	0 (0.00)	
not documented	223 (54.4)	220 (62.9)	3 (5.0)	
Was fertility sparing treatment offered by a GO?				<***0.001*** ^c^
No	235 (57.3)	218 (62.3)	17 (28.3)	
Yes	175 (42.7)	132 (37.7)	43 (71.7)	
Treatment Plan—first definitive step				0.28 ^c^
Chemo	23 (5.6)	22 (6.3)	1 (1.7)	
Chemo/RT	34 (8.3)	30 (8.6)	4 (6.7)	
Radiation	7 (1.7)	7 (2.0)	0 (0.00)	
Surgery	346 (84.4)	291 (83.1)	55 (91.7)	
Days between contact and treatment	18.5 [8.0, 35.0]	19.0 [8.0, 34.0]	17.0 [7.0, 56.5]	0.44 ^b^

Statistics are presented as median [P25, P75], N (column %). *p*-values: b = Wilcoxon Rank Sum test, c = Pearson’s chi-square test.

**Table 3:** REI referral and fertility treatments.

Factor	N	Referral Placed (N = 60)
Was REI visit attended?	60	
No		11 (18.3)
Yes		49 (81.7)
Patient offered fertility treatment?	49	
No		4 (8.2)
Yes		45 (91.8)
Did patient elect to undergo fertility testing?	45	
No		17 (37.8)
Yes		28 (62.2)
Did patient elect to undergo fertility preservation?	45	
No		24 (53.3)
Yes		21 (46.7)
Fertility treatment: medication preservation	21	
No		16 (76.2)
Yes		5 (23.8)
Fertility treatment: oocyte preservation	21	
No		10 (47.6)
Yes		11 (52.4)
Fertility treatment: embryo preservation	21	
No		21 (100.0)
Fertility treatment: ovarian tissue preservation	21	
No		14 (66.7)
Yes		7 (33.3)
Pregnancy after fertility preservation?	21	
No		19 (90.5)
Yes		2 (9.5)

Statistics are presented as median [P25, P75], N (column %).

**Table 4:** Pregnancy outcomes.

Pregnancy after onc diagnosis/intervention (regardless of REI referral)?	410				0.99 ^d^
No		392 (95.6)	334 (95.4)	58 (96.7)	
Yes		18 (4.4)	16 (4.6)	2 (3.3)	
Days between contact and last follow-up	410	1405.0 [547.0, 2767.0]	1405.0 [541.0, 2818.0]	1427.5 [747.5, 2525.0]	0.77 ^b^

Statistics are presented as median, N (column %). *p*-values: b = Wilcoxon Rank Sum test, d = Fisher’s exact test.

**Table 5:** Mixed logistic model predicting referral placement.

Factor	OR	95% CI	*p*-Value
Living children		-Reference-	
No living children	11.46	(2.28, 57.61)	***0.004***
Past medical history		-Reference-	
No past medical history	6.69	(1.71, 26.23)	***0.007***
Male Gyn Onc		-Reference-	
Female Gyn Onc	3.80	(1.04, 13.85)	***0.043***

OR: odds ratio, CI: confidence interval.

## Data Availability

Our de-identified data are available upon request to the corresponding author.

## References

[1] Woodruff T.K. (2007). The Emergence of a New Interdiscipline: Oncofertility. Cancer Treat. Res..

[2] Ethics Committee of the American Society for Reproductive Medicine (2018). Fertility preservation and reproduction in patients facing gonadotoxic therapies: An Ethics Committee opinion. Fertil. Steril..

[3] Holman D.A. (2019). Fertility Preservation in Gynecologic Cancer. Semin. Oncol. Nurs..

[4] Kohn J.R., Kashi P.K., Acosta-Torres S., Beavis A.L., Christianson M.S. (2021). Fertility-Sparing Surgery for Patients with Cervical, Endometrial, and Ovarian Cancers. J. Minim. Invasive Gynecol..

[5] Nezhat C., Roman R.A., Rambhatla A., Nezhat F. (2020). Reproductive and Oncologic Outcomes after Fertility-Sparing Surgery for Early Stage Cervical Cancer: A Systematic Review. Fertil. Steril..

[6] Bastings L., Baysal O., Beerendonk C.C.M., Braat D.D.M., Nelen W.L.D.M. (2014). Referral for Fertility Preservation Counselling in Female Cancer Patients. Hum. Reprod..

[7] Goodman L.R., Balthazar U., Kim J., Mersereau J.E. (2012). Trends of Socioeconomic Disparities in Referral Patterns for Fertility Preservation Consultation. Hum. Reprod..

[8] Banerjee R., Tsiapali E. (2016). Occurrence and Recall Rates of Fertility Discussions with Young Breast Cancer Patients. Support. Care Cancer.

[9] Hartup L.A., Go V.-A., Robinson R.D. (2024). Standardized Reproductive Endocrinology and Infertility Consultation for Pediatric and Adolescent Oncology Patients. J. Adolesc. Young Adult Oncol..

[10] Stewart K., Campbell S., Frumovitz M., Ramirez P.T., McKenzie L.J. (2021). Fertility Considerations Prior to Conservative Management of Gynecologic Cancers. Int. J. Gynecol. Cancer.

[11] Nitecki R., Woodard T., Rauh-Hain J.A. (2020). Fertility-Sparing Treatment for Early-Stage Cervical, Ovarian, and Endometrial Malignancies. Obs. Gynecol..

[12] Kipling L.M., Shandley L.M., Mertens A.C., Spencer J.B., Howards P.P. (2024). The Use of Fertility Treatments among Reproductive-Aged Women after Cancer. Fertil. Steril..

[13] Wang Y., Chen L., Ruan J.Y., Cheung W.Y. (2016). Discussions about Reproductive and Sexual Health among Young Adult Survivors of Cancer. Cancer Med..

[14] Hohmann C., Borgmann-Staudt A., Rendtorff R., Reinmuth S., Holzhausen S., Willich S.N., Henze G., Goldbeck L., Keil T. (2011). Patient Counselling on the Risk of Infertility and Its Impact on Childhood Cancer Survivors: Results from a National Survey. J. Psychosoc. Oncol..

[15] Winship A.L., Alesi L.R., Sant S., Stringer J.M., Cantavenera A., Hegarty T., Requesens C.L., Liew S.H., Sarma U., Griffiths M.J. (2022). Checkpoint Inhibitor Immunotherapy Diminishes Oocyte Number and Quality in Mice. Nat. Cancer.

[16] Macklon K.T., De Vos M. (2024). Cryopreservation of ovarian tissue for fertility preservation in breast cancer patients: Time to stop?. Reprod. Biomed. Online.

[17] Floyd J.L., Campbell S., Rauh-Hain J.A., Woodard T. (2021). Fertility Preservation in Women with Early-Stage Gynecologic Cancer: Optimizing Oncologic and Reproductive Outcomes. Int. J. Gynecol. Cancer.

[18] Lambertini M., Blondeaux E., Bruzzone M., Perachino M., Anderson R.A., de Azambuja E., Poorvu P.D., Kim H.J., Villarreal-Garza C., Pistilli B. (2021). Pregnancy After Breast Cancer: A Systematic Review and Meta-Analysis. J. Clin. Oncol..

[19] Natsuhara K.H., Chien A.J. (2024). Impact of Systemic Therapy on Fertility in Women with Early-Stage Breast Cancer. Curr. Breast Cancer Rep..

[20] United States Cancer Statistics (USCS) (2019). Gynecologic Cancer Incidence, United States—2012–2016.

[21] Riggan K.A., Rousseau A.C., DSouza K.N., Woodward K.T., Lue J., Phelan S.M., Allyse M.A., Shenoy C.C. (2023). Patient Perceptions of Body Mass Index Restrictions Limiting Fertility Care for Women with High Body Mass Index. Reprod. Biomed. Online.

[22] Kaye L., Sueldo C., Engmann L., Nulsen J., Benadiva C. (2016). Survey Assessing Obesity Policies for Assisted Reproductive Technology in the United States. Fertil. Steril..

[23] Quinn G.P., Vadaparampil S.T., Lee J.-H., Jacobsen P.B., Bepler G., Lancaster J., Keefe D.L., Albrecht T.L. (2009). Physician Referral for Fertility Preservation in Oncology Patients: A National Study of Practice Behaviors. J. Clin. Oncol..

[24] Carter J., Penson R., Barakat R., Wenzel L. (2012). Contemporary Quality of Life Issues Affecting Gynecologic Cancer Survivors. Hematol. Oncol. Clin. N. Am..

[25] Jemal A., Bray F., Center M.M., Ferlay J., Ward E., Forman D. (2011). Global Cancer Statistics. CA Cancer J. Clin..

[26] Lee S.J., Schover L.R., Partridge A.H., Patrizio P., Wallace W.H., Hagerty K., Beck L.N., Brennan L.V., Oktay K. (2006). American Society of Clinical Oncology American Society of Clinical Oncology Recommendations on Fertility Preservation in Cancer Patients. J. Clin. Oncol..

[27] Lawrenz B., Jauckus J., Kupka M.S., Strowitzki T., von Wolff M. (2011). Fertility Preservation in >1,000 Patients: Patient’s Characteristics, Spectrum, Efficacy and Risks of Applied Preservation Techniques. Arch. Gynecol. Obs..

[28] Kim J., Oktay K., Gracia C., Lee S., Morse C., Mersereau J.E. (2012). Which Patients Pursue Fertility Preservation Treatments? A Multicenter Analysis of the Predictors of Fertility Preservation in Women with Breast Cancer. Fertil. Steril..

[29] Wnuk K., Świtalski J., Miazga W., Tatara T., Religioni U., Olszewski P., Augustynowicz A. (2023). The Usage of Cryopreserved Reproductive Material in Cancer Patients Undergoing Fertility Preservation Procedures. Cancers.

